# Differences in the Levels of Physical Activity and Sport Habits between Men and Women in Cartagena (Spain)

**DOI:** 10.3390/sports12010028

**Published:** 2024-01-11

**Authors:** Celia Armada, Bernardino Javier Sánchez-Alcaraz, Javier Courel-Ibáñez, Eduardo Segarra-Vicens

**Affiliations:** 1 Faculty of Sport Sciences, University of Murcia, 30720 Murcia, Spain; bjavier.sanchez@um.es (B.J.S.-A.); esegarra@um.es (E.S.-V.); 2 Faculty of Sport Sciences, University of Granada, 18071 Granada, Spain; courel@ugr.es

**Keywords:** physical activity, sedentary, sport, health, healthy habits

## Abstract

Scientific evidence proves the importance of physical activity and sports in decreasing morbidity and mortality rates and health-related costs. Public and stakeholder involvement is vital in the sustainable promotion of physical activity and sports practice in local settings. The aim of this study was to identify the levels of physical activity and sports habits of the population of the city Cartagena (Spain). The short version of the International Questionnaire of Physical Activity was used (IQPA) and a virtual questionnaire on sports habits was sent to 1450 citizens. Responses from 248 people (162 men and 86 women), with ages ranging from 18 to 77 years old (average age = 41 ± 17 years old), were collected. The results showed low to moderate levels of physical activity with no considerable differences between men and women for the population of Cartagena. Women were shown to engage in higher intensity practice of physical activity, whereas men were shown to be more consistent and have a significantly higher participation rate in sports events, both those with free entry and those that require the acquisition of a ticket. Men were also shown to have a higher rate of media sport use. This information may assist in the development of effective political actions to promote physical activity and sports in local settings.

## 1. Introduction

Increased technological development, paired with the reduction in physical effort in the work environment, has led to an increase in sedentary lifestyle that is decisively influencing an increase in the prevalence of non-contagious diseases caused by poor or nonphysical activity, especially in adults and the elderly [1]. At the same time, the well-being of society, paired with scientific improvements, is increasing in the population of older people, who have a higher life expectancy; this is shown in every demographic study. This number is significantly higher in more developed countries. The estimated population of people over the age of 60 years old for the year 2050 is close to 417 million people [2]. 

To solve this problem, a new concept is being considered: “Health in all politics”, where sport and physical activity have become a powerful tool for new political ideas that governments can use at any level of decision-making to prevent health problems and increase the quality of life of any person [3,4]. Scientific evidence proves the importance of physical activity and sport as a means to prevent morbidity and reduce mortality rates. Physical activity and sport are also essential in reducing the costs linked to health problems [5,6]. 

According to a 2015 World Health Organization report [6,7,8], inactivity is defined as poor or no physical activity in tasks where individuals must be physically active. This refers to time spent at work, transport, domestic chores, and social time. This problem can be made worse when a social lifestyle with no regular sport-related physical activity is added. Inactivity is the fourth highest risk factor for mortality in the world, according to the Ministry of Health; this represents 6% of deaths for those people who have an energetic expense lower than 1000 kcal per week. Furthermore, it is estimated that, in eastern countries, 13.4% of all deaths could be avoided if physically inactive people changed their lifestyle and became active [8,9]. 

This paradigm change, boosted by technological development and the COVID-19 pandemic, has increased the sedentary habits of young people during their leisure time (36.4%), and this sedentary leisure time is longer for women (40.3%) than for men (32.3%) [5]. The difference between men and women was around 14 percentage points in 2020 [10]. In lower social classes, the difference ranges from 20.4% to 46.3% [10]. This led to the creation of the “Worldwide Action Plan of Physical Activity 2018–2030)” [2]. With the objective of creating “More active people for a healthier planet”, physical activity becomes a key action to reach the objectives of increased sustainable development (OSD) by the year 2030 [4,5,6,7,8,9,10,11]. 

However, the European Health Survey of Spain (2020) [12] demonstrated that 36.4% of the population occupied almost all their free time with sedentary activities (32.3% of men and 40.3% of women) and, furthermore, 50.7% do not perform any type of physical activity during their social time on any day of the week. This percentage is slightly higher in women, and it progressively increases as age increases. Regarding active mobility, 47.2% of the population walks every day to move from one place to another, and only 7.8% of the population uses a bicycle to travel from one place to another, with a higher percentage in men and young people [13,14]. Furthermore, according to the Spanish Survey of Sports Habits (2022), approximately 6 out of 10 people (57.3%) aged 15 years old or over practiced a sport during the last year, 23.8% of people practiced every day, and 52.5% practiced less than once a week. Of the survey respondents, 56.2% practiced less than once a month, and 56.7% practiced less than once every 3 months. 

In Spain, the majority (73%) of adults over 40 years old are physically inactive or sedentary during their leisure time [15]. This percentage increases up to 83% in the female population [14]. These data are alarming as low levels of physical activity (PA) have a negative impact on people’s health and quality of life, including high sanitary costs (EUR 992 million annually) [16,17]. Increments of physical activity, strength, and cardiorespiratory fitness are an effective preventive measure against premature mortality and cancer, among other diseases [18,19]. Furthermore, from a psychological standpoint, people who regularly perform physical exercise are perceived to be healthier, have lower levels of stress, and have better moods than those who are sedentary [20]. 

In 2016, the Spanish Agency of Health in Sport Protection conducted a study examining physical activity and pathological prevalence in the Spanish population [21] and found that, generally, the Spanish population has a positive perception of their physical and psychological state. However, the study showed very different results; sedentary people were found to have a far worse perception of their health than people who are physically active. The population that considered their health to be bad, very bad, or more or less bad reduced from approximately 35% to 13% as the amount of physical activity performed increased. A total of 65% of sedentary people believed that their health was good; this number increased to 87% for the people who practiced physical activity. A significant difference could be observed between the two genders, with 77.8% of men having a good opinion of their health compared to 70.9% of women; women had a higher negative opinion than men, 7.8% versus 5.6%, respectively. Because of this, understanding the sports habits and levels of physical activity of the population can serve as a useful reference when applying programs encouraging adherence to a sport. Therefore, the objective of this study was to determine the levels of physical activity and the sports habits of the population of Cartagena (Spain). We hypothesized that there are statistically significant differences regarding physical activity levels and sports habits according to gender. 

## 2. Materials and Methods

This is an observational, descriptive, cross-sectional study using self-administrated questionaries to collect representative data on the physical activity levels and sport habits of men and women age ≥ 18 years of age residents in Cartagena city (Spain). An estimate sample size of 106 people was set to meet a 95% confidence interval with a 10% margin of error for a population of 213,000. 

Physical activity was measured using the short version of the International Questionnaire of Physical Activity (IQPA) [22,23]. According to the results, individuals were classified into engaging in low, moderate or high levels of physical activity [24]. 

Sports habits were measured by a validated virtual survey. The instrument combined dichotomous and 4-point Likert scale questions to evaluate the sports habits by means of people’s participation in sport events, sports media use, and expense on and consumption of sport, among others. 

**Figure 1:**
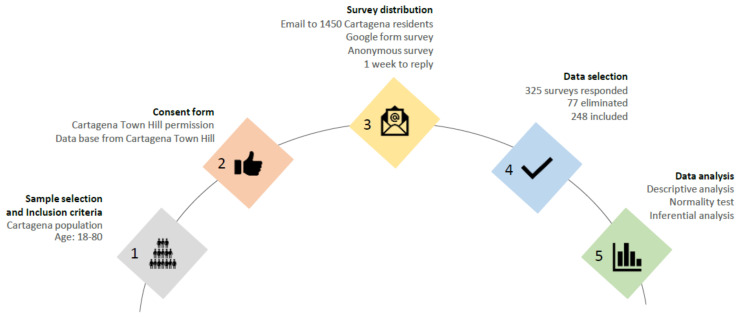
Research process steps.

A virtual recruitment was conducted by email through a database from the town hall of Cartagena. Participants provided their signed informed consent before enrollment. A dedicated Google form including the questionnaires was used to collect the data anonymously. Figure 1 shows the research process.

## 3. Data Analysis

Descriptive analyses were computed to obtain means and standard deviations (SD). The Chi-Square test and z-scores were used to compare the differences of physical activity levels and sports habits between men and women. The adjusted standardized residuals (ASR) were calculated to identify significant differences. The effect size (ES) was estimated using the Crammer’s V, where values of 0.1 represented a small effect, 0.3 a medium effect, and 0.5 a large effect [25]. The level of significance was set at *p* < 0.05. Calculations were carried out in SPSS v.24.0 for Macintosh.

## 4. Results

The questionaries were sent to 1450 people. Responses from 248 people (162 men and 86 women), who were aged 18 to 77 years old (average age = 401 ± 17 years old) and residents of Cartagena (Murcia, Spain), were collected.

There were no differences in physical activity levels between men and women (Table 1). Overall, 40% of the sample reported moderate levels of physical activity, 30% low levels, and 30% high levels.

Sports habits are described in Table 2. Men were significantly more likely to sign up to sports activities than women, with approximately 60% of the sample showing interest in town hall initiatives that promote physical activity.

When it comes to health, almost half of the men performed a physical medical exam prior to performing sports practice this year, compared to approximately 30% of women, which constitutes a significant difference. About half of the sample admitted using technological devices during physical activity practice, with a higher percentage in men. However, there were no significant differences. The participation in sports events was low, with almost half of the participants admitting to never participating in popular sports events, and only between 10 and 15% of the participants having participated in more than five events, with no significant difference between men and women. On the other hand, a considerable difference was found between men and women in sport media use, where approximately half of men followed at least one sport through television at least twice a week, compared to only 20% of women. Regarding monthly expenses on physical and sports activities, both men and women reported investing EUR 10 to 60 monthly. Finally, both men and women believed that the organization of a sport event would mainly increase the sport-related prestige and image of the city.

## 5. Discussion

The aim of this study was to find out the levels of physical activity and sports habits of the population of Cartagena (Spain). We found higher levels of physical activity compared to the ones obtained by the 2021 Spanish Survey of Health for the Region of Murcia, where 23.07% had moderate levels of physical activity (40% in our sample) and 67.87% had low levels of sports practice (30% in our sample). Our results are closer to the ones obtained by the 2022 Survey of Sports Habits in Spain [25], that indicated 52.5% of people engaged in sports practice almost once per week and 56.2% of the population practiced sport at least once a month. These data are alarming given that low levels of physical activity have a negative impact on people’s health and quality of life, involving a high sanitary cost.

Physical activity levels were similar between men and women. This is in contrast to the 2022 Sports Habits Survey of Spain [25], where sports practice was 11.3% higher in men (63.1%) than in women (51.8%), regardless of the frequency. Similarly, a student-based work from Zaragoza [26] showed larger physical activity differences between men and women (60.53% and 38.75%, respectively). The 2020 Europe Survey of Health by the National Institution of Statistics [12] for the Region of Murcia stated that high-intensity physical activity practice was mostly performed by men (12.75%) compared to women (5.17%) but that moderate practice was more common in women (27.13%) than in men (19.23%). However, seven out of ten people reported lower levels of physical activity, with no differences between men (68.03%) and women (67.70%). Facilitating access to a federative license or a subscription to a sport entity may increase adherence to sports practice. According to the 2022 Spanish Survey of Sports Habits, 52.8% of people use a specific sport facility, which is 7.5% higher than in 2020. The development of sports facility planning by the public authorities should be considered one of the main policies for reducing the high rate of physical inactivity [18].

Attendance at sports events may motivate physical activity endorsement. More than half of women admitted to never attending sports events, whilst almost 25% of men attend at least once a month. These results concur with the 2022 Spanish Sports Habits Survey, where 84% of men reported regular sport event attendance. However, the use of different instruments may explain these large differences. Attendance at sports events may be influenced by ticket cost. Indeed, free sports events were highly appreciated by both men (69.8%) and women (58.2%), with a frequency of attendance between one and five times a year (37.7% for men and 39.5% for women). These findings encourage public and stakeholder actions towards facilitating access to sport events by reducing entry costs.

Interestingly, the majority of people use sports media frequently (88.3% of men and 69.7% of women), with similar results compared to the 2022 Spanish Sports Habits Survey (82.3% of men, 62.6% of women). However, this high use of media seems to not be associated with higher levels of physical activity. Thus, while media serve as a main entertainment channel, the current offer and strategies appear insufficient in promoting physical activity and sport adherence. 

The use of technological devices during physical activity practice was common among men (52.5% use at least one device) but more infrequent in women (57.0% do not use them). Among regular physical activity practitioners (e.g., fitness center users), seven out of ten people used technological devices (61.9% of men and 38.1% of women) [27]. Nonetheless, the fact that sporty people use technology does not mean that the use of technology would increase physical activity levels. Indeed, the value of technology in promoting physical activity is in discussion, as many devices lack formal validation, and are abandoned at a high rate [19]. 

Finally, the organization of a sport event in the city seems to increase the sport-related prestige and image of the location, as recognized by more than the half of the respondents [28]. The positive socioeconomic impact of sports event organization has been acknowledged previously (e.g., the GP of Europe of Formula 1 in Valencia [29], the Tour of France in Utrecht [30], La Vuelta Ciclista [31,32]). In addition, the organization of big events may serve to stimulate parallel sport activities and promote physical activity among attendees.

This study has certain limitations that must be considered when interpreting the results. The sample size is local and limited to the population of Cartagena. Furthermore, the use of self-reported questionnaires may distort the true levels of physical activity compared to technology-based methods. 

## 6. Conclusions

A high percentage of the population of Cartagena have a moderate to low level of physical activity, without noticeable differences between men and women. Public and stakeholder actions to increase the level of physical activity could involve reducing ticket costs, increasing access to facilities, and promoting the organization of National and International sports events.

## Figures and Tables

**Table 1:** Sex differences of physical activity levels.

		Sex						X^2^	*p*	ES
		Men			Women					
		N	%	ASR	N	%	ASR			
Physical activity levels	Low	51	31.7	0.1	26	31.0	−0.1	0.557	0.757	0.048
	Moderate	69	42.9	0.5	33	39.3	−0.5			
	High	41	25.5	−0.7	25	29.9	0.7			

Note: N = Number, % = Percentage; ASR = Adjusted standardized residuals; X^2^ = Chi square; *p* = Significance level; ES = Effect size estimated by Cramer’s V.

**Table 2:** Sex differences in terms of sports habits.

		Sex						X^2^	*p*	ES
		Men			Women					
		N	%	ASR	N	%	ASR			
Are you subscribed to any sport entity?	Yes	93	57.4	2.5	35	40.7	−2.5	6.281	0.012 *	0.149
	No	69	42.6	−2.5	51	59.3	2.5			
Are you interested in any campaign by the town hall of Cartagena to increase your sport-related practice?	Yes	92	56.8	−0.6	52	60.5	0.6	0.312	0.577	0.035
	No	70	43.2	0.6	34	39.5	−0.6			
Have you performed a medical exam prior to performing physical activity this year?	Yes	73	45.1	2.6	24	27.9	−2.6	6.942	0.008 *	0.167
	No	89	54.9	−2.6	62	72.1	2.6			
Do you use a technological device to monitor your physical activity?	Yes	85	52.5	1.4	37	43.0	−1.4	2.005	0.157	0.090
	No	77	47.5	−1.4	49	57.0	1.4			
How often do you participate in popular sports events?	1 or 2 events	47	29.0	1.4	18	20.9	−1.4	3.019	0.389	0.110
	3 or 4 events	15	9.3	−0.6	10	11.6	0.6			
	More than 5 events	24	14.8	0.7	10	11.6	−0.7			
	Never	76	46.9	−1.3	48	55.8	1.3			
Do you attend sports events where you must buy a ticket?	Once a month	38	23.5 a	3.8	4	4.7 a	−3.8	16.402	0.001 *	0.257
	Once every 2 months	12	7.4 a,b	0.8	4	4.7 a,b	−0.8			
	Between 1 and 5 times a year	55	34.0 b	−0.9	34	39.5 b	0.9			
	Never	57	35.2 b	−2.4	44	51.2 b	2.4			
Do you assist at sports events where the entry is free?	Once a month	37	22.8	1.7	12	14.0	−1.7	6.056	0.109	−156
	Once every two months	15	9.3	1.3	4	4.7	−1.3			
	Between 1 and 5 times a year	61	37.7	−0.3	34	39.5	0.3			
	Never	49	30.2	−1.8	36	41.9	1.8			
Do you follow sport through media?	2 times a week or more	80	49.4 a	4.7	16	18.6 a	−4.7	27.202	<0.001 *	0.331
	Once a month	27	16.7 b	−0.2	15	17.4 b	0.2			
	Between 1 and 3 times a month	36	22.2 b,c	−2.0	29	33.7 b,c	2.0			
	Never	19	11.7 c	−3.6	26	30.2 c	3.6			
How much do you spend a month on sport-related activities?	Less than 10 euros	37	22.8	−1.1	25	29.1	1.1	1.385	0.847	0.075
	Between 10 and 60 euros	86	53.1	1.0	40	46.5	−1.0			
	Between 60 and 120 euros	27	16.7	−0.2	15	17.4	0.2			
	Between 120 and 240 euros	10	6.2	0.1	5	5.8	−0.1			
	More than 240 euros	2	1.2	0.0	1	1.2	0.0			
In your opinion, the creation of a sport event in your city would…	Produce an increase in the investments of the location	36	22.2	0.4	17	19.8	−0.4	7.961	0.093	0.179
	Improve the solidarity and hospitality of the residents regarding visitors	14	8.6	−0.2	8	9.3	0.2			
	Increase the sports-related prestige of the location	52	32.1	−2.5	41	47.7	2.5			
	Improve the exterior image of the location	51	31.1	1.9	18	20.9	−1.9			
	Do nothing. I believe it would not have any impact	9	5.6	1.2	2	2.3	−1.2			

Note: N = Number, % = Percentage; ASR = Adjusted standardized residuals; X^2^ = Chi square; *p* = Significance level; ES = Effect size estimated by Cramer’s V. * Significant differences (*p* < 0.05). a–c = Differences in column percentages according to Bonferroni.

## Data Availability

Data are unavailable due to privacy restrictions.
